# Dynamic changes in the T cell receptor repertoire during treatment with radiotherapy combined with an immune checkpoint inhibitor

**DOI:** 10.1002/1878-0261.13082

**Published:** 2021-09-01

**Authors:** Åsa Kristina Öjlert, Daniel Nebdal, Igor Snapkov, Vibeke Olsen, Joel Kidman, Victor Greiff, Jonathan Chee, Åslaug Helland

**Affiliations:** ^1^ Department of Cancer Genetics Institute for Cancer Research Oslo University Hospital Norway; ^2^ Department of Immunology University of Oslo Norway; ^3^ National Centre for Asbestos Related Diseases Institute of Respiratory Health University of Western Australia Perth WA Australia; ^4^ School of Biomedical Sciences University of Western Australia Perth WA Australia; ^5^ Department of Clinical Medicine University of Oslo Norway; ^6^ Department of Oncology Oslo University Hospital Norway

**Keywords:** abscopal response, immunotherapy, non‐small cell lung cancer, radiotherapy, T cell receptor sequencing

## Abstract

Previous studies have indicated a synergistic effect between radiotherapy and immunotherapy. A better understanding of how this combination affects the immune system can help to clarify its role in the treatment of metastatic cancer. We performed T cell receptor (TCR) sequencing on 46 sequentially collected samples from 15 patients with stage IV non‐small cell lung cancer, receiving stereotactic body radiotherapy combined with a programmed cell death ligand‐1 (PD‐L1) inhibitor. TCR repertoire diversity was assessed using Rényi diversity curves and the Shannon diversity index. TCR clones were tracked over time. We found decreasing or stable diversity in the best responders, and an increase in diversity at progression in patients with an initial response. Expansion of TCR clones was more often seen in responders. Several patients also developed new clones of high abundance. This seemed to be more related to radiotherapy than to immune checkpoint blockade. In summary, we observed similar dynamics in the TCR repertoire as have been described with immunotherapy alone. In addition, the occurrence of new unique clones of high abundance after radiotherapy may indicate that radiotherapy functions as a personalized cancer vaccine.

## Graphical abstract

Radiotherapy and immunotherapy have been proposed to work synergistically in the treatment of cancer. We used T cell receptor (TCR) sequencing to monitor the immunological response to combined stereotactic radiotherapy and PD‐L1 blockade in patients with metastatic non‐small cell lung cancer. Specifically, we investigated possible mechanisms of action and the potential of the TCR repertoire as a dynamic biomarker.

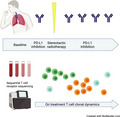

**Abbreviations**
ICI: immune checkpoint inhibitor
PBMCs: peripheral blood mononuclear cells
PD‐1: programmed cell death protein‐1
PD‐L1: programmed cell death ligand‐1
SBRT: stereotactic body radiotherapy
TCR: T cell receptor

## 1 Introduction

Immune checkpoint inhibitors (ICIs) have recently been implemented into the standard care of non‐small cell lung cancer (NSCLC) and have shown ability to improve both short‐ and long‐term prognosis [1, 2, 3, 4, 5, 6]. However, the majority of patients with advanced NSCLC do not benefit from ICIs. Various treatment combinations have been investigated with the aim of enhancing patient outcomes. While ICIs have been successfully combined with chemotherapy [7, 8], the role of radiotherapy in this context is not yet clear. Secondary analysis of the KEYNOTE‐001 trial showed better progression free survival (PFS) and overall survival (OS) for patients who received any radiotherapy prior to immunotherapy [9]. In several studies investigating the safety of combining radiotherapy with an ICI, this combination was well tolerated [10, 11]. Stereotactic body radiotherapy (SBRT) combined with pembrolizumab was compared prospectively to pembrolizumab alone in the PEMBRO‐RT trial, with an overall response rate (ORR) at 12 weeks of 18% in the control arm and 36% in the experimental arm [12]. Interestingly, the largest benefit was seen in patients with PD‐L1‐negative tumors.

Though radiotherapy is mainly regarded as a localized treatment, a response in tumor lesions outside the radiation field, an abscopal response, is sometimes observed. This effect is thought to be immune‐mediated in that radiotherapy induces the release of tumor associated antigens (TAAs) which are taken up by antigen‐presenting cells and presented to naïve T cells in the lymph nodes. Activated T cells are then released to the blood stream and may target tumor cells both within and outside the radiation field [13]. Radiotherapy also triggers the release of pro‐inflammatory cytokines and damage‐associated molecular patterns (DAMPs), resulting in a more general activation of the immune system [14]. Still, immunosuppressive mechanisms in the tumor microenvironment are thought to be the main reason why abscopal responses are rare and are the basis for combining radiotherapy with immunotherapy [15].

How, and to what degree, radiotherapy might potentiate the effect of ICIs needs to be further studied. An increased understanding of this is important, both to optimize the design of future clinical trials and to identify predictive biomarkers. High throughput TCR sequencing is a tool used to examine how therapy changes antigen‐specific T cell immunity. Profiling the TCR repertoire in serial samples reveals features such as clonal expansions, the persistence and turnover of T cell clones. Recent studies demonstrate that TCRs clustered by sequence similarity potentially target similar antigens [16, 17]. By characterizing the TCR repertoire before and during treatment, we sought to find indications of whether response was mainly an effect of immune checkpoint blockade, possibly enforced by the pro‐inflammatory effects of radiotherapy, or if radiotherapy leads to a significant release of new antigens and thereby functions as an *in situ* vaccine. Lastly, early changes in the TCR diversity during treatment with ICIs have been correlated with response [18], and hence, we were also interested to see if this applies when radiotherapy is combined with a PD‐L1 inhibitor.

## 2 Materials and methods

### 2.1 Patients

Patients with advanced NSCLC were eligible for inclusion if they had received previous treatment with a platinum doublet and had an Eastern Cooperative Oncology Group (ECOG) performance status of 0 or 1, age > 18 years, a life expectancy of > 3 months, no previous treatment with a PD‐1 / PD‐L1 inhibitor, adequate organ function, a tumor lesion suitable for radiotherapy treatment and measurable disease according to Response Evaluation Criteria in Solid Tumors (RECIST), not including the irradiated lesion(s). Patients with an EGFR mutation or an ALK translocation could participate if they had previously received treatment with a tyrosine kinase inhibitor. Patients with brain metastases were eligible if these were treated/stable.

### 2.2 Trial design and treatment

The Combinatory ImmunoTherapy‐1 (ComIT‐1) trial is a multi‐center phase II trial combining SBRT and the PD‐L1 inhibitor atezolizumab. The primary endpoint is toxicity, and the secondary endpoints include response rates, overall survival, safety and tolerability, PFS, abscopal effects and quality of life. Exploratory endpoints include immunological response, tumor evolution, and dynamics in the tumor microenvironment. Twenty‐one patients were included in the study before it closed for inclusion in December 2020. Inclusion slowed down when immune checkpoint blockade became available for more NSCLC patients outside clinical trials, and it therefore closed before all planned 30 patients were enrolled.

**Fig. 1:**
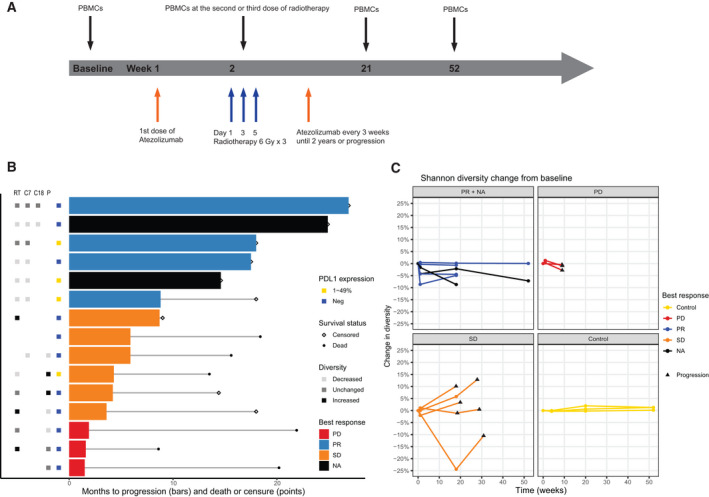
T cell receptor diversity and clinical outcomes. (A) Study overview. (B) Clinical outcomes, programmed death ligand‐1 (PD‐L1) expression on tumor cells and T cell receptor diversity for all patients (*n* = 15). Diversity refers to change in Shannon diversity from baseline and was defined as unchanged if there was < 1% deviation from baseline. (C) Change in Shannon diversity over time plotted per patient. For one of the patients with stable disease, baseline was missing, and radiotherapy is therefore used as the starting point. PBMCs, peripheral blood mononuclear cells; PD, progressive disease; PR, progressive disease; SD, stable disease; RT, radiotherapy; C7, cycle 7; C18, cycle 18; P, progression.

Atezolizumab was administered at day 1 of every 21‐day cycle at a dose of 1200 mg and was continued for up to two years or until no clinical benefit. SBRT 6 Gy x 3 was administered to tumor lesion(s) of at least 2 ccm (median 53.3 ccm, range 2.1–465.2 ccm) in volume approximately 1 week after the first dose of immunotherapy (Fig. 1A). The radiotherapy dose was chosen based on results from preclinical trials investigating the abscopal response [19].

The study has been approved by the regional ethics committee (South East REC 2017/1845) and was performed in accordance with the standards of The Helsinki Declaration. Written informed consent was collected from all patients before enrollment.

### 2.3 Study assessments

The tumor response was assessed according to RECIST version 1.1 every 2‐3 months until progression or stop of treatment. Adverse events were reported according to the common terminology criteria for adverse events (CTCAE) version 4.0. Plasma / serum samples were collected at baseline, during radiotherapy, at every evaluation time point and at progression. Peripheral blood mononuclear cells (PBMCs) were collected at baseline, at the second or third dose of radiotherapy, at cycle 7, at cycle 18 and at progression or 2 years of follow‐up. PBMCs were only collected from patients included at Oslo University Hospital.

### 2.4 T cell receptor sequencing

Sequential TCR sequencing was performed on 46 blood samples from 15 patients, included between September 2018 and March 2020. PBMCs were isolated from blood by density gradient centrifugation using Lymphoprep (Alere Technologies, Oslo, Norway) and cryopreserved in liquid nitrogen. DNA was isolated using the QIAamp DNA blood mini kit (Qiagen, Hilden, Germany). The CDR3 regions of T cell receptor β chains were amplified using the hsTCRB v3 kit (Adaptive Biotechnologies, Seattle, WA, USA) as deep survey. During DNA extraction one of the samples (108 Radiotherapy) was contaminated by DNA from the baseline sample from the same patient. Comparisons between baseline and radiotherapy for this patient are therefore uncertain. Quantification of the libraries was performed with Qubit HS DNA (Thermo Fisher, Waltham, MA, USA) and the size distribution controlled using TapeStation with D1000 and High Sensitivity D1000 Screen Tape (Agilent Technologies, Santa Clara, CA, USA). Sequencing was performed on an Illumina NextSeq 500/550 Mid Output flowcell (Illumina, San Diego, CA, USA), loading 1.5 pm of pooled libraries and 20‐40% PhiX. An overview of number of sequenced genomes, unique nucleotide sequences and unique productive nucleotide sequences per sample can be found in Table S1.

PBMC TCR sequencing data from a study including three healthy individuals sampled at eight time points over a year was downloaded from the immune ACCESS repository immunoSEQ (Adaptive Biotechnologies; https://clients.adaptivebiotech.com/pub/healthy‐adult‐time‐course‐TCRB) [20].

### 2.5 Statistical analyses

All statistical analyses were performed in r version 4.0.2 [21] using the LymphoSeq [22], vegan [23], ggplot2 [24], and igraph [25] packages. Diversity was calculated with the renyi function from the vegan package, based on TCR CDR3 amino acid sequence and with default parameters. To control for variations in library size, 70 000 TCRs were picked and summed from each sample before Rényi diversity [26] was calculated. This was repeated with 500 permutations and the results averaged. Rényi diversity was defined as.

$$H_{\alpha }\left(p\right)\;=\;\frac{1}{1‐\alpha }\;\text{ln}\sum_{i=1}^{S}p_{i}^{\alpha }$$

where *p_i_* is the proportion of species *i*, *S* is the number of species and α modulates the sensitivity to species abundances [27]. α = 0 gives the number of unique TCR clones in the sample, the species richness. Gradually, more weight is put on the abundance of clones as α increases. The output of the renyi function when α = infinity is defined as the negative natural logarithm of the frequency of the most abundant clone. Rényi diversity curves hold more information than univariate diversity indices [28]. However, sometimes univariate indices are more convenient to use. The case α = 1 is not defined by the Rényi formula, but by the Shannon diversity index [29]. It is influenced by both the richness and abundance of clones and is a commonly used measure to describe TCR diversity. We used Rényi diversity curves to look for overall patterns in diversity and Shannon diversity to compare samples from the same patient over time.

The igraph package was used to make networks based on amino acid sequence similarity and a Levenshtein distance of 1. TCRs with frequency < 0.001 were not included in the network analysis.

Given the small sample size, the study design was exploratory and statistical tests to compare groups were not performed.

## 3 Results

### 3.1 Subject characteristics

A total of 46 samples from 15 patients receiving atezolizumab combined with SBRT in the ComIT‐1 trial were included in the analyses. All patients had previously treated advanced NSCLC, five patients had received > 1 systemic treatment lines prior to inclusion, 9 patients were male and median age was 64.7 years (range 50.3–79.2). Four patients had 1%–49% PD‐L1‐positive tumor cells and 11 were PD‐L1 negative. Patient 114 had an EGFR mutation in exon 19. When data were collected in December 2020, median survival time was not yet reached and median PFS was 5.9 months (95% CI 4.2‐not reached). Median follow‐up time, calculated as median observation time after initiation of immunotherapy for patients still alive at the end of follow‐up, was 18 (range 9‐27) months. Four patients had a partial response (PR), six stable disease (SD) and three progressive disease (PD) as best response. In two of the patients, best response could not be evaluated. However, these two patients had not progressed when data were collected after 18 and 25 months of follow‐up, respectively, and were therefore considered good responders. Clinical and molecular characteristics are summarized in Table 1.

**Table 1:** Clinical and molecular characteristics. AD, adenocarcinoma; SCC, squamous cell carcinoma; LCNEC, large cell neuroendocrine carcinoma; ADSq, adenosquamous carcinoma; BR, best response; BR rt, best response of the irradiated lesion(s). PD‐L1 refers to PD‐L1 expression on tumor cells. RT target refers to localization of the tumor lesion(s) treated with stereotactic radiotherapy.

Patient	Histology	PD‐L1	RT target	BR	BR rt	Progression status	PFS (months)	Survival status	OS (months)
101	AD	Negative	Adrenal gland	PR	PR	Censored	26.9	Censored	26.9
102	AD	Negative	Pleura	PD	SD	Progression	1.9	Dead	21.9
103	AD	Negative	Lung	NA	NA	Censored	24.9	Censored	24.9
104	LCNEC	1%–49%	Lung	PR	PR	Censored	18	Censored	18
105	SCC	1%–49%	Lung	SD	PR	Progression	4.3	Dead	13.5
106	AD	Negative	Lung	SD	NA	Progression	4.2	Censored	14.4
107	AD	Negative	Mediastinum	SD	PR	Progression	5.9	Dead	15.6
108	AD	Negative	Lung	SD	SD	Progression	3.6	Censored	18
109	NSCLC	1‐49%	Adrenal gland	PR	PR	Progression	8.8	Censored	18
110	AD	Negative	Lung	PR	PR	Censored	17.5	Censored	17.5
111	AD	Negative	Liver	PD	SD	Progression	1.6	Dead	8.6
112	AD	1%–49%	Brain	NA	CR	Censored	14.6	Censored	14.6
113	ADSq	Negative	Lung	PD	SD	Progression	1.5	Dead	20.2
114	AD	Negative	Lung	SD	SD	Progression	5.9	Dead	18.4
115	AD	Negative	Lung	SD	SD	Progression	8.7	Censored	9

### 3.2 TCR diversity and patient outcome

Diversity is a measure of the number of unique TCRs (richness) and their similarity in frequency [27]. A decrease in diversity is seen when a subset of TCR clones expand and dominate and has been correlated with response to treatment in some studies with ICIs as monotherapy. We assessed diversity using Rényi diversity curves and compared this to patient outcome. No association was observed between diversity at baseline and best response or PD‐L1 status (Fig. S1). In sequential samples from the same patient, we saw more variation in the abundance of clones than in the number of unique TCRs (richness), when controlling for library size (Fig. S2).

To follow diversity in the same patient over time, we used the Shannon diversity index and defined this as increased or decreased if it deviated > 1% from baseline. We found that the best responders had either decreased (*n* = 4) or unchanged (*n* = 2) diversity at radiotherapy. The same pattern was seen for all the following time points (Fig. 1B+C). In the six patients with SD or PD, the changes in diversity varied more, both between patients and over time in the same patient. Of note, only one patient with SD and no patients with PD had decreased diversity at radiotherapy. Five patients who initially had SD had samples taken at progression. In four of these, there was an increase in diversity at the time of progression, compared to the most recent sample prior to progression (Fig. 1C). The fifth patient did not have increased diversity at the time of radiological progression. However, this patient continued treatment beyond progression and had an increase in diversity at the time when treatment was stopped due to no clinical benefit. There was no clear association between the size of the radiotherapy target and change in diversity (Fig. S3).

### 3.3 The majority of new TCRs were not similar to TCRs found at baseline

**Fig. 2:**
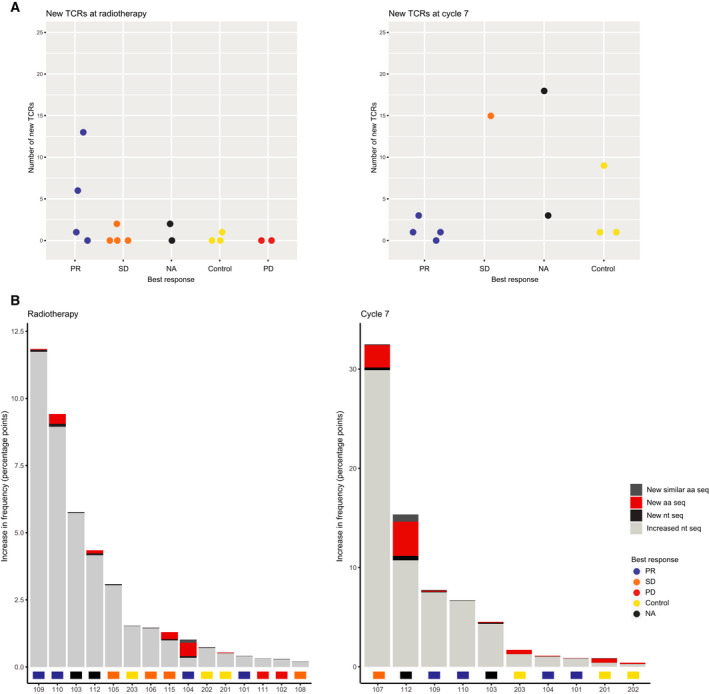
New T‐cell clones of high abundance. (A) Number of new T cell receptors (TCRs) among the 100 most frequent TCRs at radiotherapy and cycle 7. Each dot represents a patient or a healthy control. (B) Total increase in frequency of the top 100 most abundant TCRs that were new or increased at radiotherapy and cycle 7, compared to baseline. The color indicates if the increase happened in new TCRs (New aa seq), new TCRs with an amino acid sequence that differed by only one amino acid from an existing TCR (New similar aa seq), TCRs present at baseline (Increased nt seq) or TCRs with new nucleotide sequences but identical amino acid sequences as existing clones (New nt seq). aa seq, amino acid sequence; nt seq, nucleotide sequence.

As a common model to explain abscopal radiotherapy response includes release of tumor antigens followed by activation of naïve T cells, we assessed if new TCR clones of high abundance could be detected after treatment. We compared the frequency of the 100 most abundant TCRs at radiotherapy and cycle 7 to baseline. Samples were available from both baseline and radiotherapy for 12 patients. TCRs that were not detected at baseline were found among the top 100 most frequent clones at radiotherapy in 3 of 4 patients with PR, 1 of 4 patients with SD, none of the two patients with PD and one patient where best response could not be evaluated (Fig. 2A). As it typically takes about a week from the first exposure to a new antigen until maximal clonal expansion [30], we thought that it would be less likely to find new clones of high abundance in those sampled already at the second dose of radiotherapy. One patient with SD, one with PR and one with unknown best response (patients 101, 103, and 105) had samples taken at the second, instead of the last, dose of radiotherapy and none of these had new clones of high abundance.

Seven patients had samples collected at both baseline and cycle 7. Of these, six had new TCR clones among their top 100 most frequent clones. These results were compared to dynamics seen over time in three healthy individuals, sampled 1 and 5 months after baseline. At 1 month, one of the healthy controls had a new TCR, while all three controls had new TCRs among their top 100 most frequent clones 5 months after baseline (Fig. 2A).

New clones developing during treatment are not necessarily targeting new antigens. To get an impression of whether new clones were most likely directed towards antigens already recognized by other T cells, we compared the amino acid sequence of new TCRs to those present at baseline. If the sequence of the new TCR differed from existing TCRs by only one amino acid (a Levenshtein distance of 1), we assumed that there was a high likelihood for that these would bind to the same antigen [17]. Both at radiotherapy and at cycle 7 the majority of new TCRs with high abundance were different from TCRs present at baseline. The 5 patients who had new TCRs among their top 100 most frequent clones at radiotherapy had 24 new TCRs in total, of which only two were similar to existing TCRs in the same patient. These two were found in the same patient, and that patient also had 11 new TCRs that were unrelated to existing clones. 6 of 7 patients had new TCRs at cycle 7. Three of these had new clones that were similar to existing TCRs, but all of those also had new clones that differed by > 1 amino acid from those found at baseline. In total, 9 of 41 TCRs were similar to clones present at baseline.

In addition to TCRs with similar amino acid sequence targeting the same antigen, there can be multiple TCRs with unique nucleotide sequences coding for the same amino acid sequence. Just as with very similar TCRs, we found it likely that these represent a broadening of the repertoire directed at already recognized antigens. New nucleotide sequences, corresponding to already present amino acid sequences, were found for the 100 most frequent TCRs at radiotherapy and cycle 7 in all patients. However, these did not contribute much to the total increase in frequency in the top clones. In Fig. 2B, the total increase in frequency for new and existing TCRs, compared to baseline, is shown per patient. Though several patients had new TCRs among their top 100 most abundant clones, expansion of existing clones contributed most to the total increase.

### 3.4 New clones after one dose of ICI rarely remained abundant

Four of the patients who had new TCRs among their 100 most frequent clones at radiotherapy had samples taken at cycle 7. At that time point, all new TCRs had disappeared or dramatically decreased in frequency, with the exception of one TCR found in patient 112 (Table S2). This particular clone had increased from 0.08% to 2.4% of the repertoire and had thereby become the most abundant clone. At cycle 7, patient 112 stands out with a considerable increase in new TCRs (Fig. 2B). Interestingly, this patient had received additional radiotherapy, both concomitantly with the SBRT administered in ComIT‐1 and one month later, for symptom relief. The patient also developed treatment‐induced colitis before cycle 7, but not until two months after radiotherapy, making it unlikely that the most abundant clone at cycle 7 was related to this. However, we cannot know if new TCRs observed in this patient were targeting tumor or self‐antigens. As a subset of these had either identical or similar amino acid sequence as TCRs present at baseline, it seems plausible that these represent a broadening of the repertoire against previously recognized tumor antigens. The TCR that was new at radiotherapy and had become the most abundant at cycle 7, was not similar to other TCRs in the baseline, radiotherapy or cycle 7 samples from this patient, implying that the specificity for this clone might be a new antigen.

### 3.5 Expanded TCR clones decreased at progression

**Fig. 3:**
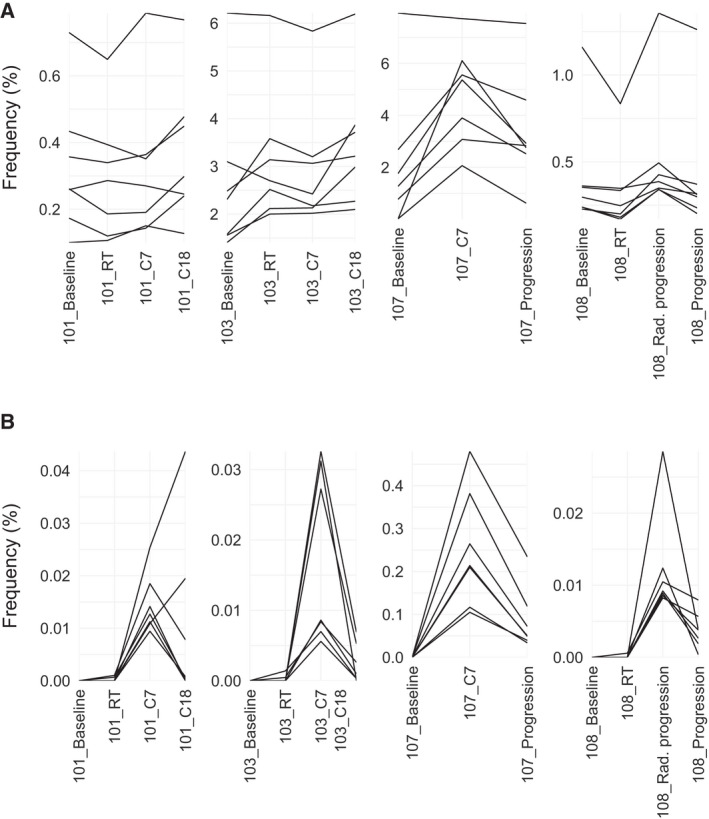
T cell clones tracked over time. (A) The seven most abundant T cell receptor clones at the time point corresponding to cycle 7 followed over time in two patients who had still not progressed at cycle 18 (patients 101 and 103), one patient who progressed after cycle 7 (patient 107), and one patient who had radiological progression at cycle 7 but who continued treatment until no clinical benefit (patient 108). (B.) The seven most abundant new T cell receptor clones at the time point corresponding to cycle 7 tracked over time in the same patients as in (A). Rad. progression, radiological progression.

Four of five patients with an initial response had an increase in diversity at progression, suggesting that expanded clones had decreased at that time point (Fig. 1C). To verify this, we tracked TCR clones over time in 4 patients (Fig. 3). Two of these patients were still progression free after 1 year of follow‐up (patients 101 and 103), one progressed after cycle 7 (patient 107) and one had radiological progression at cycle 7, but continued treatment until no clinical benefit and had a new sample taken when treatment was stopped (patient 108).

In general, the frequencies of the most expanded clones varied a lot between patients (Table S1, Fig. S4). We picked the seven most abundant of all clones and the seven most abundant of new clones at cycle 7 and followed these over time (Fig. 3A+B). In patient 107, who progressed after cycle 7, most of these TCRs had decreased at progression and none had increased. In patient 101 and 103, the majority of clones that were high at cycle 7 continued to increase or remained stable at cycle 18. Only patient 101 had new clones of high abundance at cycle 7 that had not decreased or disappeared at cycle 18. In patient 108, the most abundant clones at baseline (Fig. S4) did not decrease until this patient quit treatment due to no clinical benefit. The most abundant clones at radiological progression also decreased at this time point. It then seems like the TCR dynamics correlated better with clinical than radiological measures of response in this patient.

## 4 Discussion

Reports from preclinical studies, and from a limited number of clinical studies, have raised the hope that radiotherapy may increase the fraction of patients who benefit from ICIs [31]. In this exploratory study, we examined dynamics in the TCR repertoire during treatment with a PD‐L1 inhibitor combined with SBRT, with the aim to better understand the background for a synergistic effect between these treatments.

TCR diversity has been proposed as a predictive and dynamic biomarker in patients treated with ICIs as monotherapy. We found decreased or stable diversity in the best responders, and an increase in diversity at progression in patients with an initial response. Low diversity has been associated with more advanced disease, and decreasing diversity with rapid progression, in patients with NSCLC receiving other treatments than ICIs [32]. While CTLA‐4 inhibitors seem to increase diversity regardless of patient outcome [33, 34], dynamics observed under treatment with PD‐1/PD‐L1 inhibitors have been more variable. When an association between diversity and response to PD‐1/PD‐L1 inhibitors has been found, a decrease in diversity has been more beneficial [35, 36]. It makes sense that PD‐1/PD‐L1 blockade, and growing disease burden outside the context of checkpoint blockade, both lead to expansion of tumor‐related clones, and thereby a decrease in diversity. Our findings suggest that the combination of radiotherapy and a PD‐L1 inhibitor affects the diversity in responders in a similar way as PD‐1/PD‐L1 blockade alone and that changes in diversity can be of help to monitor response.

Lung tumors often have high mutational burden, and thereby many neo‐antigens that can trigger an immune response, but the mutational landscape varies between patients and often also within the same tumor [37, 38]. This has been a challenge in the development of therapeutic cancer vaccines [39]. Radiotherapy may act as a highly individualized tumor vaccine, by causing exposure of tumor‐related peptides [40]. Both decreasing diversity and expansion of specific TCR clones have been observed when radiotherapy has been given alone [41, 42]. However, these changes do not seem to persist over time, a problem that ICIs might help to overcome. We found new TCRs of high abundance both at radiotherapy and at cycle 7. As the full TCR repertoire cannot be sampled, there is a possibility that clones defined as new rather were not previously detected clones. The risk of defining a rare clone as new is affected by library size. In this study, this was especially relevant when comparing patients to healthy controls, as the controls were from a study using extra deep sequencing. By concentrating on the most abundant TCRs, we hoped to reduce this problem, but especially TCRs with low abundance that rapidly increased during treatment would still be at risk of being identified as new.

We did not find new clones of high abundance in samples taken at the second dose of radiotherapy, but found several such clones in those sampled at the last dose of radiotherapy. At both of these time points, there should have been enough time to observe clonal expansion after the first dose of ICI, and this was also seen in two of the patients sampled already at the second dose of radiotherapy, but not enough time to achieve clonal expansion of naïve T cells for those sampled early during radiotherapy [30]. This supported the hypothesis that new clones were mainly a result of radiotherapy, and not of immune checkpoint blockade. In this context, it was also interesting to see that one patient, receiving two series of palliative radiotherapy in addition to SBRT, had a new clone at radiotherapy that was not similar to previously detected clones and that expanded to become the most abundant at cycle 7.

At cycle 7, most patients had new clones of high abundance. The number and frequency of these varied between patients suggesting that they might be of importance in some, but not all, responders. Some new clones differed by only one amino acid from existing clones. This could mean that naïve T cells got exposed to antigens previously recognized by other T cells, but where these had not managed to remove all tumor cells expressing this antigen. New nucleotide sequences coding for the same amino acid sequence as existing clones were common in low frequencies. Though these might represent a broadening of the repertoire against the most immunogenic neo‐antigens, sequencing errors could also contribute to the observed TCR convergence [43].

Studies examining the TCR repertoire when immunotherapy is combined with radiotherapy are still few. Formenti *et␣al*. [44] investigated TCR dynamics during combinatorial treatment with SBRT and CTLA‐4 blockade and found expansion of some TCR clones and contractions of others in responders, while little change was observed in nonresponders or in those with stable disease. New clones were also detected and in one of the patients it was confirmed that two of these were tumor‐specific. To our knowledge, this is the first study examining TCR dynamics in patients receiving radiotherapy combined with a PD‐1/PD‐L1 inhibitor. It is limited by its small sample size and the approach has therefore been exploratory / hypothesis generating. Another limitation is that we lacked information on antigen specificity for the identified TCRs, and we can therefore not be sure that new and expanding clones were tumor‐related. The study supports the hypothesis that radiotherapy can lead to activation of naïve T cells, mimicking the effects of a cancer vaccine, but more studies are needed to clarify this. We also saw expansion of existing clones, but cannot tell if this was solely a consequence of the ICI or if pro‐inflammatory effects of radiotherapy helped potentiate the immune response. A large proportion of the participants were PD‐L1 negative. This group of patients is thought to have more to gain by adding radiotherapy, and it will be interesting to see if ongoing studies can confirm this.

## 5 Conclusions

Emergence of new highly abundant TCR clones after radiotherapy may indicate that this treatment functions as an *in situ* cancer vaccine, potentiated by immunotherapy. TCR diversity holds potential as a dynamic biomarker and could be especially useful for early identification of nonresponders and as a complement to radiological assessments in cases with suspected pseudoprogression.

## Conflict of interest

Åslaug Helland has received financial support and/or study drug from Roche, AstraZeneca, Ultimovacs and BMS, in association with clinical studies.

## Author contributions

ÅH designed the study. VO prepared the libraries for the T cell receptor sequencing. ÅÖ, DN, and IS analyzed and visualized the data. ÅÖ, IS, JK, VG, JC, and ÅH interpreted the data. ÅÖ prepared the first draft of the manuscript, and all authors revised and approved the final version of the manuscript.

### Peer Review

The peer review history for this article is available at https://publons.com/publon/10.1002/1878‐0261.13082.

## Supporting information


**Fig S1.** Rényi diversity according to PD‐L1 status and best response.
**Fig S2.** Change in Rényi diversity from baseline to radiotherapy, cycle 7 and progression
**Fig S3.** Change in Shannon diversity according to volume and response of the irradiated lesion(s) and Shannon diversity plotted against total lymphocyte count.
**Fig S4.** Cumulative frequency of the seven most abundant T cell receptor clones per sample and the seven most abundant clones at baseline tracked over time.

**Table␣S1.** Sample overview.
**Table␣S2.** New T cell receptor clones found among the 100 most frequent clones at radiotherapy and cycle 7.

## Data Availability

The data that support the findings of this study are available from the corresponding author (asa.kristina.ojlert@rr-research.no) upon reasonable request.
