# Supplementary material for: Dynamic changes in the T cell receptor repertoire during treatment with radiotherapy combined with an immune checkpoint inhibitor
Source: Mol Oncol. 2021 Sep 1;15(11):2958–68. doi: 10.1002/1878-0261.13082 (PMC8564644; doi:10.1002/1878-0261.13082)

Supplementary figure S1. Rényi diversity at baseline and at radiotherapy. No association was observed between diversity at baseline, or diversity after one dose of atezolizumab, and PD-L1 status or best response.

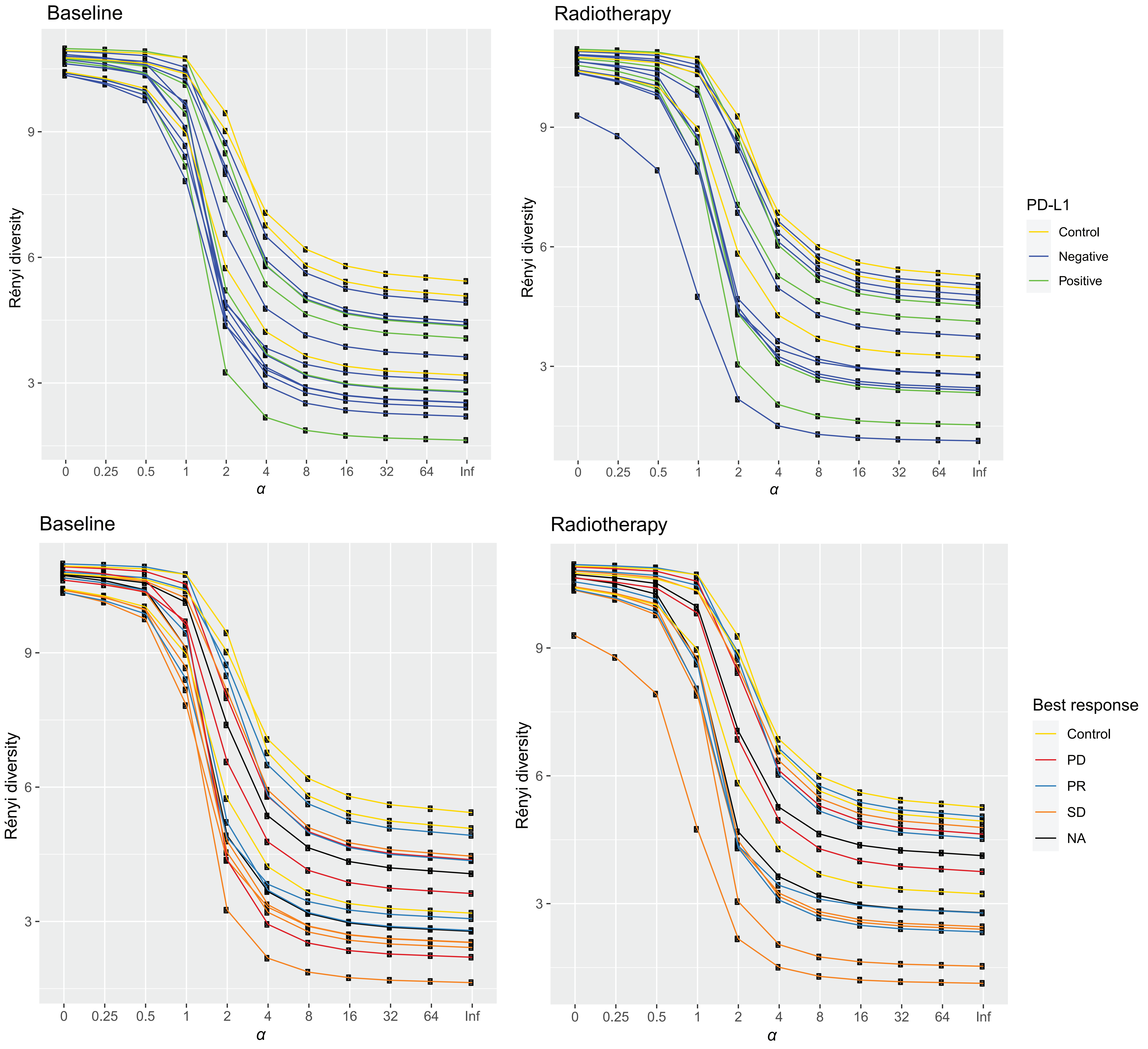

Supplementary figure S2. Change in Rényi diversity from baseline to radiotherapy, cycle 7 and progression. On the x axis 1 corresponds to Shannon diversity. Color refers to patient ID and the large dots to the right show change of area under the Rényi curve per patient.

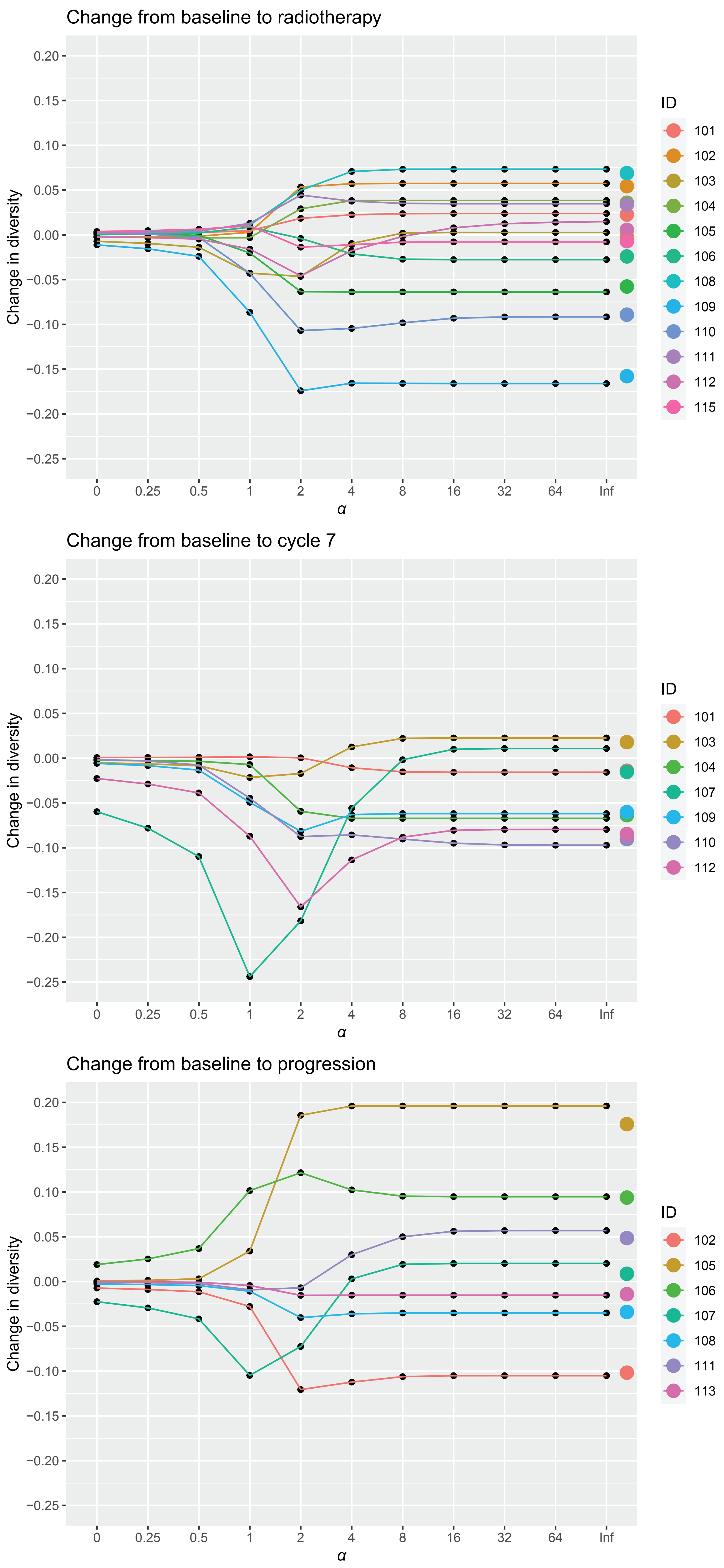

Supplementary figure S3A. Change in Shannon diversity per patient according to best response of the irradiated lesion(s). B. Change in Shannon diversity according to volume of the irradiated lesion(s). C. Shannon diversity plotted against lymphocyte count at the time when the sample was collected.
PR: partial response, CR: complete response, SD: stable disease. Baseline diversity was missing for one of the patients and percentage change from the radiotherapy timepoint is therefore shown.

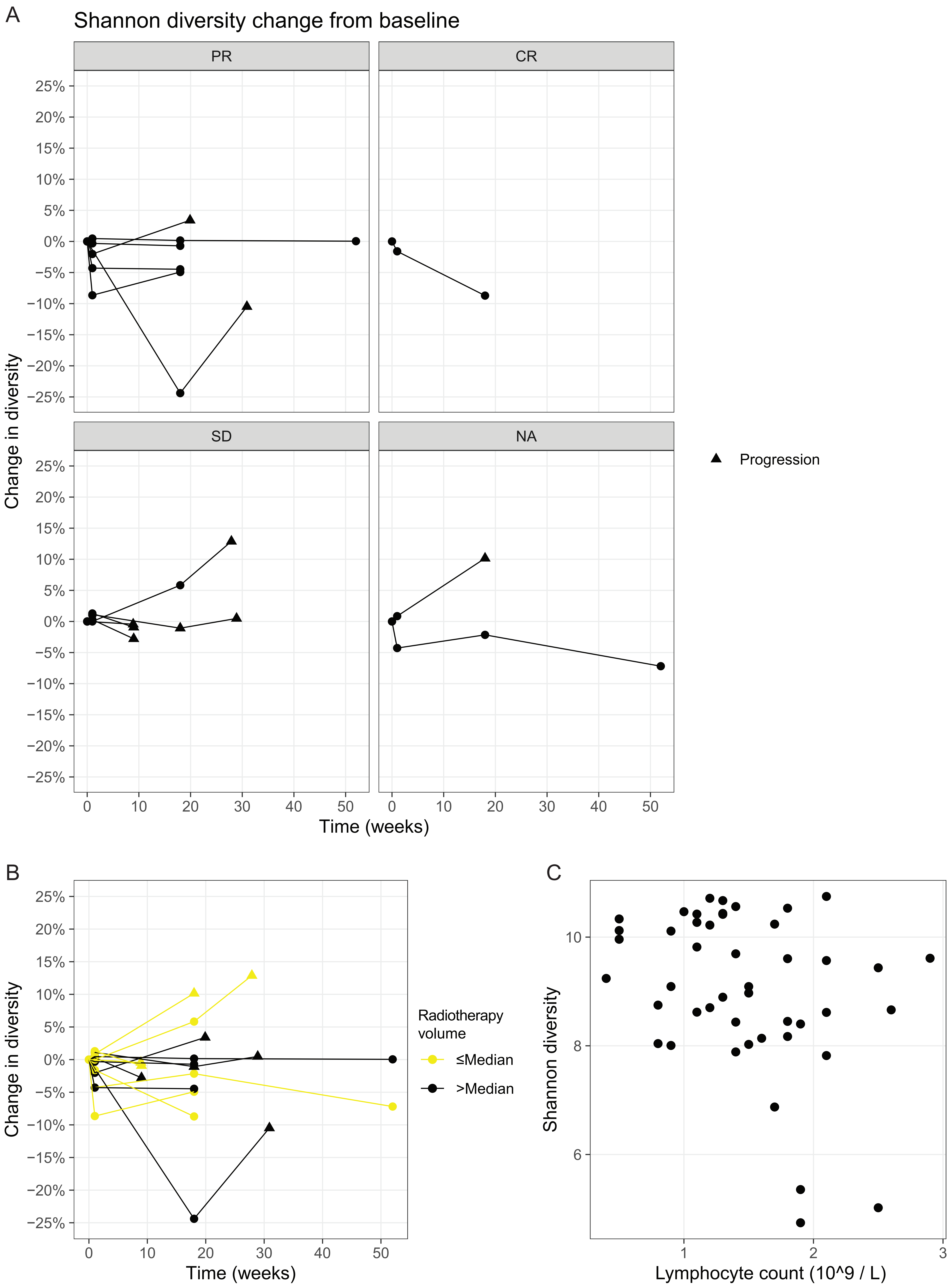

Supplementary figure S4A. Cumulative frequency of the seven most abundant TCR clones per sample. B. The seven most abundant clones at baseline tracked over time per patient.

A

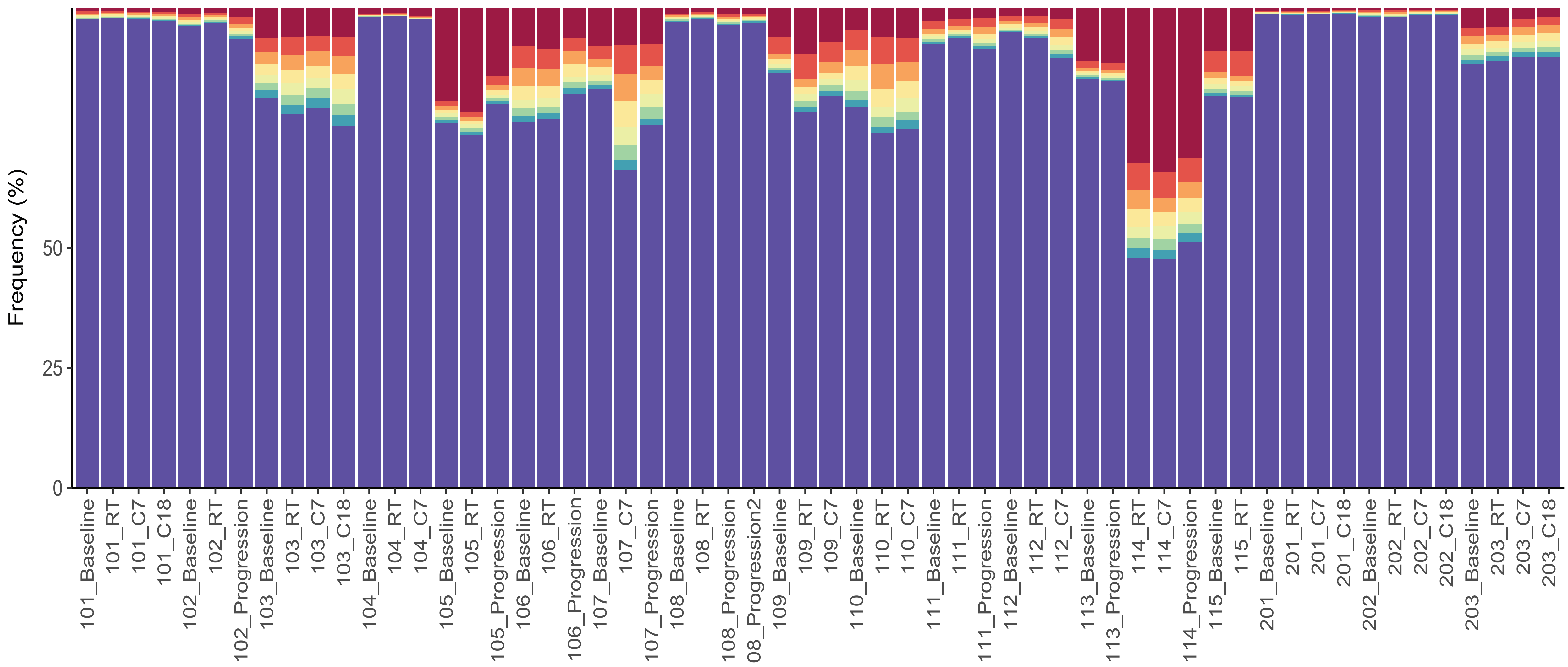

B

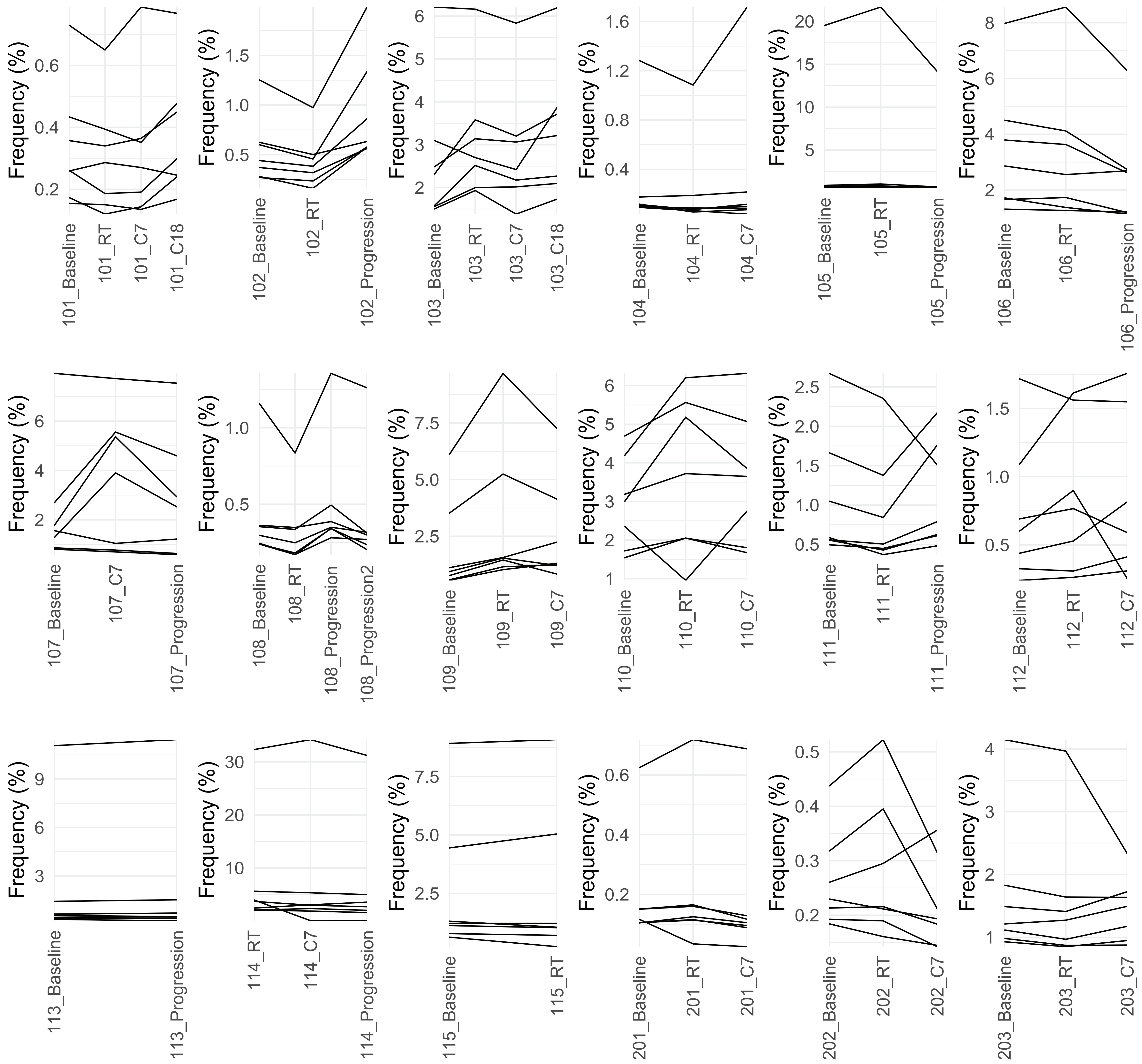

Supplement: Supplementary file 1 — Fig S1. Rényi diversity according to PD‐L1 status and best response. Fig S2. Change in Rényi diversity from baseline to radiotherapy, cycle 7 and progression Fig S3. Change in Shannon diversity according to volume and response of the irradiated lesion(s) and Shannon diversity plotted against total lymphocyte count. Fig S4. Cumulative frequency of the seven most abundant T cell receptor clones per sample and the seven most abundant clones at baseline tracked over time. [file MOL2-15-2958-s002.pdf]
