# Supplementary material for: Dynamic changes in the T cell receptor repertoire during treatment with radiotherapy combined with an immune checkpoint inhibitor
Source: Mol Oncol. 2021 Sep 1;15(11):2958–68. doi: 10.1002/1878-0261.13082 (PMC8564644; doi:10.1002/1878-0261.13082)
Supplement: Supplementary file 2 — Table␣S1. Sample overview. Table␣S2. New T cell receptor clones found among the 100 most frequent clones at radiotherapy and cycle 7. [file MOL2-15-2958-s001.pdf]

Supplementary table S1. Sample overview.

Samples		Unique Nucleotide Sequences	Unique Productive Nucleotide Sequences	Total Number of Genomes	Top Productive Sequence* (%)
**101**	**Baseline**	232310	190095	415207	0.73
**101**	**RT**	210991	173182	358653	0.65
**101**	**C7**	195774	160282	336951	0.79
**101**	**C18**	193498	158083	314350	0.77
**102**	**Baseline**	385262	319150	577694	1.17
**102**	**RT**	294720	244919	453400	0.90
**102**	**Progression**	285376	236970	521904	1.82
**103**	**Baseline**	292365	244259	501400	6.15
**103**	**RT**	272755	228108	510490	6.11
**103**	**C7**	250753	208393	513636	5.77
**103**	**C18**	190687	158391	364226	6.15
**104**	**Baseline**	383667	310857	520150	1.27
**104**	**RT**	313766	255129	443442	1.08
**104**	**C7**	311547	252988	429568	1.71
**105**	**Baseline**	75114	62268	167712	19.43
**105**	**RT**	65370	54148	137682	21.59
**105**	**Progression**	42978	35291	83043	14.10
**106**	**Baseline**	174304	143811	499572	7.89
**106**	**RT**	159008	131365	467772	8.48
**106**	**Progression**	138867	114102	300256	6.23
**107**	**Baseline**	232965	186953	417062	7.47
**107**	**C7**	134120	108040	469287	7.33
**107**	**Progression**	157863	127090	361978	7.16
**108**	**Baseline**	282192	226481	495195	1.16
**108**	**RT**	254136	204369	424552	0.83
**108**	**Progression**	222186	178689	392149	1.36
**108**	**Progression2**	202892	161518	329572	1.26
**109**	**Baseline**	177415	148906	346644	5.75
**109**	**RT**	162344	136300	355241	9.24
**109**	**C7**	186527	156409	385941	6.85
**110**	**Baseline**	179514	148890	593632	4.34
**110**	**RT**	168770	139574	477115	6.17
**110**	**C7**	173554	143603	545350	6.30
**111**	**Baseline**	210660	175962	465688	2.66
**111**	**RT**	202205	169198	441128	2.35
**111**	**Progression**	148118	123668	307583	2.16
**112**	**Baseline**	91621	74329	141303	1.72
**112**	**RT**	114191	92606	187874	1.60
**112**	**C7**	64805	52425	126279	2.31
**113**	**Baseline**	148947	120788	227430	10.93
**113**	**Progression**	185464	150170	289645	11.34
**114**	**RT**	26768	21265	230876	32.21
**114**	**C7**	36186	29043	220881	34.10
**114**	**Progression**	47474	38319	269981	31.12
**115**	**Baseline**	80918	66678	190094	8.88
**115**	**RT**	86834	71684	192671	9.02
**201**	**Baseline**	650834	520470	21206561	0.62
**201**	**RT**	599018	479170	20873908	0.72
**201**	**C7**	560631	445325	31359887	0.68
**201**	**C18**	510964	407558	21257715	0.49
**202**	**Baseline**	390654	325208	15441351	0.44
**202**	**RT**	351616	293333	12657870	0.52
**202**	**C7**	252872	207898	18241272	0.36
**202**	**C18**	340672	280348	23400452	0.39
**203**	**Baseline**	302882	242081	18511871	4.10
**203**	**RT**	286201	228158	18841525	3.92
**203**	**C7**	276276	218939	26294377	2.31
**203**	**C18**	282522	223024	27418353	1.87

* Percentage of the full repertoire taken up by the most abundant TCR.
RT: radiotherapy, C7: cycle 7, C18: cycle 18. Patient 108 continued treatment beyond radiological progression, and had a new progression sample taken when the treatment was stopped (108 Progression2). In controls (201-203) RT, C7 and C18 correspond to 1 month, 5 months and1 year after baseline.

Supplementary table S2. Abundance of new T cell receptors among the 100 most frequent clones at radiotherapy and cycle 7.Only patient 112 had a new clone that was among the 100 most frequent clones at both time points. At cycle 7 this was the most abundant clone.

Patient	TCR β CDR3 amino acid sequence	Count at radiotherapy	Frequency (%) at radiotherapy	Count at cycle 7	Frequency (%) at cycle 7
**101**	CASSPDRKQGGTEAFF	-	-	70	0.03
**103**	CASSARPYNEQFF	-	-	117	0.03
**103**	CASSSRGGYEQFF	-	-	134	0.03
**103**	CASSQVRSGELFF	-	-	140	0.03
**104**	CASSDEGAQNIQYF	69	0.02	-	-
**104**	CASGLVLRPSYNSPLHF	78	0.02	-	-
**104**	CASSPSTGTYEQYF	79	0.02	-	-
**104**	CASSSGTGLFGELFF	89	0.02	-	-
**104**	CASSLGYRTGRNEQYF	111	0.03	-	-
**104**	CASSLQGNTYNEQFF	125	0.03	-	-
**104**	CASLAYSGNTIYF	155	0.04	-	-
**104**	CASSVTGSSTDTQYF	165	0.05	-	-
**104**	CASSEGGYYGYTF	260	0.07	-	-
**104**	CASSPAYSTHEQYF	753	0.21	-	-
**104**	CATSDPTGSSPGWDEQFF	80	0.02	1	0.00
**104**	CANHGNTEAFF	94	0.03	2	0.00
**104**	CASSFTGLEQYF	174	0.05	2	0.00
**104**	CASSEQPGTGYEQYF	-	-	196	0.06
**107**	CSARDPEALAGQVNEQFF	-	-	208	0.05
**107**	CASTVAGVRTEAFF	-	-	246	0.06
**107**	CASSTRDLPYGYTF	-	-	253	0.06
**107**	CASSRLAYGEQFF	-	-	269	0.07
**107**	CASSDPTVSYEQYF	-	-	282	0.07
**107**	CAISESGGIGQPQHF	-	-	299	0.08
**107**	CASSEGGQPQHF	-	-	319	0.08
**107**	CASTAITGYGYTF	-	-	344	0.09
**107**	CASRPGLNLGETQYF	-	-	409	0.11
**107**	CASSPESGLFGANVLTF	-	-	455	0.12
**107**	CASQL0YSNQPQHF	-	-	817	0.21
**107**	CASSVGFLAGITDTQYF	-	-	833	0.21
**107**	CASSQDQVGRGVVDTQYF	-	-	1030	0.26
**107**	CASSGTSGEANTGELFF	-	-	1486	0.38
**107**	CAWSISDIMNTEAFF	-	-	1869	0.48
**109**	CAWSRDLRLNQPQHF	91	0.03	-	-
**109**	CASINLKAANYGYTF	3	0.00	115	0.04
**109**	CASSEAPGYTIYF	-	-	202	0.06
**109**	CASSMGNGYTF	1	0.00	292	0.09
**110**	CASSPPTEVYTEAFF	95	0.02	-	-
**110**	CASSSTWTDDFYGYTF	97	0.02	-	-
**110**	CASSLKGEGKLFF	144	0.04	-	-
**110**	CASSEYGVVRDTEAFF	239	0.06	-	-
**110**	CASSWDGSGELFF	452	0.11	-	-
**110**	CASSFGTGEQPQHF	459	0.11	-	-

Patient	TCR β CDR3 amino acid sequence	Count at radiotherapy	Frequency (%) at radiotherapy	Count at cycle 7	Frequency (%) at cycle 7
**112**	CASRAGLWMGYTF	61	0.04	-	-
**112**	CASSQGTSGVLYEQYF	-	-	60	0.06
**112**	CASSLVWDDQETQYF	1	0.00	61	0.06
**112**	CASSLGTGENTEAFF	-	-	71	0.07
**112**	CASSWDPPGTEAFF	-	-	72	0.07
**112**	CAISEPGERPYNEQFF	-	-	73	0.07
**112**	CASSLAANEQFF	-	-	73	0.07
**112**	CASSLLVVNYGYTF	-	-	77	0.07
**112**	CASSYSGQGYEQYF	1	0.00	85	0.08
**112**	CASSPPGGTDTQYF	4	0.00	93	0.09
**112**	CSGRGTEAFF	-	-	99	0.10
**112**	CASSEDGVGNTGELFF	2	0.00	101	0.10
**112**	CASSLGQGVGELFF	-	-	103	0.10
**112**	CASSVETGGINTIYF	-	-	106	0.10
**112**	CASSPDSGGPYEQYF	-	-	108	0.10
**112**	CSASYSGYEQYF	-	-	201	0.19
**112**	CASSPRGGTDTQYF	-	-	219	0.21
**112**	CASSQELSGYEQYF	-	-	281	0.27
**112**	CASSPGGWSAFF	116	0.08	2467	2.37
**115**	CASSAPPGLPTDTQYF	83	0.05	-	-
**115**	CASSGLQVEAFF	350	0.21	-	-
